# The Melanocortin System in Inflammatory Bowel Diseases: Insights into Its Mechanisms and Therapeutic Potentials

**DOI:** 10.3390/cells12141889

**Published:** 2023-07-19

**Authors:** Antonietta Gerarda Gravina, Raffaele Pellegrino, Tommaso Durante, Giovanna Palladino, Giuseppe Imperio, Giovanbattista D’Amico, Maria Consiglia Trotta, Marcello Dallio, Mario Romeo, Michele D’Amico, Alessandro Federico

**Affiliations:** 1 Hepatogastroenterology Unit, Department of Precision Medicine, University of Campania Luigi Vanvitelli, 80138 Naples, Italy; 2 Mental Health Department, S. Pio Hospital, Via dell’Angelo, 82100 Benevento, Italy; 3 School of Geriatrics, University of Studies of L’Aquila, 67010 L’Aquila, Italy; 4 Department of Experimental Medicine, University of Campania Luigi Vanvitelli, 80138 Naples, Italy

**Keywords:** melanocortin, inflammatory bowel disease, Crohn’s disease, ulcerative colitis, α-MSH, β-MSH, ACTH, melanocortin receptors, KPV, KdPT

## Abstract

The melanocortin system is a complex set of molecular mediators and receptors involved in many physiological and homeostatic processes. These include the regulation of melanogenesis, steroidogenesis, neuromodulation and the modulation of inflammatory processes. In the latter context, the system has assumed importance in conditions of chronic digestive inflammation, such as inflammatory bowel diseases (IBD), in which numerous experiences have been accumulated in mouse models of colitis. Indeed, information on how such a system can counteract colitis inflammation and intervene in the complex cytokine imbalance in the intestinal microenvironment affected by chronic inflammatory damage has emerged. This review summarises the evidence acquired so far and highlights that molecules interfering with the melanocortin system could represent new drugs for treating IBD.

## 1. Introduction

The melanocortin system is a complex and phylogenetically ancient system of peptides, comprising α-, β- and γ-melanocyte-stimulating hormone (MSH) and adrenocorticotropic hormone (ACTH), resulting from a common protein precursor, pro-opiomelanocortin (POMC). POMC is modified in the post-translational phase and provides precursor peptides (γ-MSH, ACTH, β-lipotropins) that, when adjusted by glycosylation, amidation and acetylation, provide active mediators. In particular, the α-melanotropin group is the product of the action of the precursor convertase 2 enzyme on ACTH as an enzyme substrate [1].

Molecules belonging to the melanocortin family can interact with varying degrees of affinity at five melanocortin receptors identified and characterised to date (i.e., MC1–5R). They show different topographical features but particularly nuanced receptor affinity characteristics, except for MC2R, which, on the contrary, seems to offer an exclusive affinity towards ACTH [2]. These receptors bind various agonists with different affinities, and of these, Nle-D-Phe47 (NDP)-α-MSH is among those with the greatest potency towards MC1R, MC3R, MC4R and MC5R compared with other agonists such as α-MSH, ACTH, β-MSH or γ-MSH [3]. MC2R, on the other hand, recognises ACTH(1-24) and DPhe7-ACTH(1-24) as potent agonists [3].

The receptors are expressed in a tissue-dependent manner. In detail, the MC1R receptor is expressed in a wide range of cytotypes (fibroblasts, melanocytes, keratinocytes, neutrophils, monocytes, dendritic cells, B lymphocytes, gliocytes, endotheliocytes and neoplastic cells). It responds to ACTH and α-MSH and is mainly involved in eumelanin synthesis in melanogenesis by activating the enzyme tyrosinase. As stated before, MC2R, in contrast to the other receptors, shows a specific receptor affinity for ACTH and is mainly expressed in the adrenal cortex and adipocytes and is involved in steroid synthesis. MC3R has a widespread expression pattern in the central nervous system (CNS) and immune cells (predominantly B lymphocytes and macrophages) but is also expressed in the gut, heart and placenta. It has a role mainly related to metabolic control. MC4R is the most highly expressed receptor in the CNS and is involved in regulating energy homeostasis and feeding behaviour, and ultimately, a role in neuroprotection has also been described. Finally, MC5R has a predominantly ubiquitous distribution and has been linked to the immunomodulation of B/T lymphocyte responses and the control of exocrine glandular secretions [3,4,5].

Together, MCRs belong to the macrofamily of G-protein-coupled receptors with seven transmembrane segments, for which several ligands have also been identified (see Table 1) [6].

It has been repeatedly stigmatised that the melanocortin system may play a pivotal role in inflammatory bowel diseases (IBD) [7]. Therefore, this review aims to present studies that have evaluated the possibility of modulating the system regarding the pathogenesis and therapy of IBD, with a particular focus on melanocortin receptors.

## 2. α-MSH as a Key Melanocortin in the Modulation of Inflammatory Processes

The pathogenesis of IBD is particularly complex and far from fully elucidated. It is thought to be the product of the interaction between several pathogenic elements, including the intestinal epithelium, DNA-related factors (genetic and epigenetic), the intestinal mucosal system and the gut microbiota. In addition, psychopathological aspects have also been called into question, both as an epidemiological element of a high prevalence of mood disorders in patients with IBD (even in the absence of disease activity) [8,9] and pathogenesis [10].

The mucosal immune system, however, plays a key role [11]. In detail, mucosal-activated macrophages can produce large amounts of tumour necrosis factor (TNF), interleukin (IL) 6, IL-12 and IL-23, known proinflammatory mediators. At the same time, neutrophils can degranulate the contents of their intracellular granules. Lymphocyte cells also play a role as dendritic cells, and their migration into the mesenteric lymph nodes can present antigens to CD4^+^ T lymphocyte cells, inducing the proliferation of T helper lymphocytes (i.e., T_H_1 or T_H_17), which produce additional proinflammatory mediators (i.e., interferons, IL-17A, IL-17F, IL-22).

In the intestinal microenvironment of IBD, dysregulation of cytokines, the production of which is strongly controlled by nuclear factor kappa-light-chain-enhancer of activated B cells (NF-κB), is therefore particularly evident. This production is dramatically altered and imbalanced in favour of increased production of proinflammatory cytokines (such as TNF-α, IL-1 and IL-6), as evidenced by the increased concentration of NF-κB p65 in intestinal macrophages within intestinal biopsies from patients with IBD [12]. The expression levels of this molecule also correlated directly with disease severity [13]. NF-κB is activated in macrophages and epithelial cells of the inflamed intestinal mucosa. Among the previously mentioned cytokines, TNF and IL-1 activate metalloproteinases responsible for mucosal tissue damage and promote the differentiation of lymphocytes in the T_H_1 direction [14].

The production of TNF in all these processes has thus gained prominence in the development of biological agents directed towards IBD in both Crohn’s disease (CD) and ulcerative colitis (UC) to the extent that anti-TNF (i.e., infliximab, adalimumab, golimumab) are still among the biologics of first choice in the therapeutic management of IBD and related major extraintestinal manifestations [15,16,17].

The direct role of melanocortins in the regulation of inflammatory processes has emerged from their potential to inhibit the family of NF-κB involved in the transcriptional regulation of many genes involved in the synthesis of cytokines (especially TNF) and related receptors, chemokines and adhesion molecules [18,19,20]. 

NF-κB can exert its activity in the presence of an adaptor that gives it the potential to activate the transcriptional process (activating domain). However, some proteins in this family, such as NF-κB1,2, lack this domain. They, therefore, reside in the cytoplasm, connected to an inhibitor molecule, nuclear factor of kappa light polypeptide gene enhancer in B-cells inhibitor alpha (IkBα). IkBα can be degraded, for example, by activating a series of receptors of the inflammatory system (such as TNFR, TLR, IL-1R), which, by promoting its degradation, allow NF-κB to translocate within the nucleus and act as a transcription factor [21].

NF-κB plays a role in both innate and acquired immunity. For example, it can be induced by toll-like receptors if they use myeloid differentiation primary response gene 88 (MyD88) as an adaptor. All toll-like receptors (TLR 1-9) are potential activators of MyD88, except for TLR3 [21,22].

α-MSH is a 13-amino-acid neuroendocrine peptide that may play a vital role in these processes. For example, it may be responsible for downgrading NF-κB and inhibiting IL-8 within endotoxin-stimulated monocytes and tumour necrosis factor (TNF) [23,24,25].

The effects of α-MSH are also exerted in the lymphocyte population. Evidence has shown its potential to induce immunological tolerance mechanisms and promote the development of CD4^+^ CD25^+^ T-regulatory lymphocytes. These regulatory T cells require antigen recognition for activation, but through nonspecific TGF-β1-mediated mechanisms, they can suppress other effector T cells, thus exhibiting immunomodulatory action [13,14]. Furthermore, several cytokines such as IL-2, IFN-γ and IL-10 are under the regulation of α-MSH within antigen-induced cell proliferation. Interleukin-10 inhibits other proinflammatory cytokines such as IL-2, IFN-γ and TNF-α, and α-MSH has been shown to reduce the antigen-induced proliferation of splenic cells and nonregulatory CD4^+^-CD25^-^ lymphocytes [26]. Indeed, it appears that α-MSH may mediate the induction of TGF-β-producing T cells and suppressing the production of IFN-γ [27,28].

IBD has also been associated with TGF-β signalling. It has emerged from genome-wide association studies how certain gene variations in TGF-β may be related to an increased risk of IBD onset and how TGF-β1 deficiency is associated with very-early-onset IBD [29].

Furthermore, among the numerous pathways activated by α-MSH is the Janus kinase pathway, which has been extensively studied in IBD [11]. In detail, it was found that α-MSH, by binding its MC5R receptor in Ba/F3 lymphocytes, activates the JAK2 pathway [25].

## 3. The Receptors of the Melanocortin System: The Basis for Their Role in Peripheral and Systemic Inflammation

To understand the system’s potential in IBD, it is necessary to weigh the possibilities that identified melanocortin receptors have in peripheral and systemic inflammatory processes.

### 3.1. MC1R

MC1R is the main receptor involved in the mechanisms of melanogenesis and response to ultraviolet (UV) radiation [23,30]. Nevertheless, it has been implicated in several other homeostatic processes. Indeed, it is expressed within the vascular endothelium, acting not only in regulating vascular tone but also in preventing endothelial dysfunction following cell migration processes [31]. Several MC1R polymorphisms have been associated with a lower risk of complicated post-traumatic sepsis in preclinical models, suggesting its possible role in the post-traumatic inflammatory response [32]. In a mouse model of streptozotocin-induced diabetic retinopathy, the selective MC1R activation reduced the retinal vascular alterations and decreased the retinal proinflammatory cytokines and chemokines [33]. These anti-inflammatory actions were specifically observed in primary retinal cells from mice exposed to high glucose concentrations, with enhanced antioxidant levels and the preservation of photoreceptor integrity [34]. In mouse models, MC1R-induced activation of the transcriptional pathway (and other nerve-distributing melanocortin receptors such as MC4R) showed neuroprotective effects in neuroinflammatory processes, probably through induction and promotion of T-regulatory lymphocyte activity [35,36]. 

### 3.2. MC2R

MC2R has been reported in some inflammatory pathways. Some authors wanted to evaluate the expression profiles of melanocortin receptors about the response to adalimumab in patients with rheumatoid arthritis. It was found that the expression of MC2R, MC3R and MC4R is reduced in CD8^+^ and CD19^+^ lymphocytes in anti-TNF-responsive patients after three months of follow-up [37]. However, the precise molecular mechanism underlying this modulation is still unknown.

### 3.3. MC3R

Numerous experimental results have demonstrated the involvement of this receptor in inflammatory processes. Several molecules capable of interacting with MC3R have shown the anti-inflammatory properties of this receptor. MC3R agonists have been extensively studied in mouse models as potential therapeutic agents under the anti-inflammatory properties derived from MC3R activation. ACTH fragments capable of activating MC3R can inhibit cytokine synthesis in peritoneal macrophages, indirectly blocking neutrophilic diapedesis [38,39]. In addition, in the cardiac muscle, where macrophages express MC3R, the administration of agonists during acute myocardial infarction showed a protective role in mice, even during reperfusion, demonstrating that this protection was due to a reduction in systemic and local inflammatory markers (i.e., IL-1 and myeloperoxidase) [40]. Also, in mouse models, the same protective effects were identified in gouty arthritis [41]. The anti-inflammatory role of MC3R has also been confirmed in the context of metabolic syndrome in mice knockout for MC3R independently of weight gain [42]. This also has relevance in the context of IBD, where metabolic alterations, especially diabetes, significantly impact aspects of the disease [43].

### 3.4. MC4R

MC4R is the primary melanocortin receptor present in the central nervous system [36]. It is predominantly expressed in the cerebral cortex, hypothalamus, thalamus, spinal cord and brainstem. Its distribution in the CNS is greater than MC3R and is generally nonoverlapping. Within the ventral periventricular and premammillary nuclei as well as in the posterior hypothalamic nucleus, however, coexpression of MC3R and MC4R can be observed [44]. Due to their localisation in the brainstem, melanocortins can activate several anti-inflammatory pathways via the efferent vagal pathway, reducing the expression of proinflammatory cytokines in endotoxemia, sepsis and other inflammatory conditions [45,46,47]. The concept of immunomodulatory efferent signalling of the vagus nerve has emerged. Cholinergic neurotransmission thus plays a central role in this pathway. In particular, the “vagal inflammatory reflex” concept has been outlined in the literature over time. In detail, Tracey et al. showed in preclinical models of endotoxic shock that there is an immunomodulatory cholinergic pathway with a sensory component and a motor component. In damaged peripheral tissues, the production of cytokines and other inflammatory molecules activates the vagal afferent pathway (sensory component) as a priming of the activation of the motor component of the pathway with the release of acetylcholine in the reticuloendothelial system (in the hepatic, cardiac, splenic and gastrointestinal context) and inhibition of the production and diffusion of molecules such as TNF, IL-1 and other cytokines. These considerations relate to the activity of the melanocortin system, as after observing the positive effects of melanocortins in the prevention of haemorrhagic shock, sepsis, myocardial ischaemia and stroke, the ability of melanocortins to activate the former cholinergic pathways via MC4R was highlighted [47,48]. It has also been observed that selective MC4R agonists can increase IL-6 and IL-11 levels at the glial level. These interleukins orient astrocytic macrophages towards an anti-inflammatory phenotype [49]. Similar neuroprotective evidence was observed in further preclinical models. Of interest is the increase in MC4R expression at the alcohol-induced hippocampal–hypothalamic level. Stimulation of MC4R in this condition reduced the secretion of IL-6, IL-1 and TNF [50]. This receptor has also been implicated in cachexia processes induced by chronic, oncological or inflammatory diseases characterised by increased levels of proinflammatory cytokines. Blockade of the MC4R receptor has been shown to antagonise cytokine-induced cachexia [51].

MC4R has, over time, been associated with eating behaviour, so much so that variants of the gene coding for MC4R represent the most frequent cause of monogenic obesity. Loss-of-function mutations in this gene are associated with increased hunger and consequent weight gain [52,53]. Most melanocortin trials targeted MC4R and were obesity-focused. Obesity, however, recognises inflammation as a crucial pathogenetic element. Obesity is associated with the chronic increase of several inflammatory markers (i.e., C-reactive protein). This leads to an increased risk of cardiovascular and metabolic disorders (e.g., diabetes mellitus) [54]. Obesity can influence gut microbiota, and this relationship is also reciprocal [55]. It is also known how lipopolysaccharide from gut microbiota bacteria can induce subclinical inflammation and insulin resistance (and thus obesity) through modulation of the innate immunity toll-like receptor 4 [56]. Moreover, inflammation has also been postulated as a mediator between obesity and the increased risk it may have on several neoplastic diseases [57].

Indeed, synthetic MC4R agonists are among the most studied pharmacological agents for obesity therapy. Some examples are LY2112688, MC4-NN-0453 and AZD2820; however, results have been mixed and not definitive in nongenetic obesity [58]. On the contrary, some genetic obesity disorders related to the impairment of MC4R signalling (i.e., Bardet–Biedl syndrome) characterised by significant hyperphagia showed an excellent response to setmelanotide, a selective agonist of MC4R [59].

### 3.5. MC5R

This receptor has been mainly associated with immune-mediated inflammatory modulation and induction of the JAK2-mediated pathway [25]. MC5R has been implicated in ocular immunity, although its role in inflammatory responses is not fully elucidated. However, selective MC5R activation showed anti-inflammatory actions in diabetic retinopathy, both in vitro and in vivo [33,34], with a specific antiangiogenic activity in human retinal pigment epithelial cells [60]. Moreover, MC5R stimulation reduced hypertrophy induced by high glucose in rat cardiomyocytes [61]. Selective MC5R agonists have shown promising positive effects in immune-mediated diseases [62,63].

## 4. The Role of the Melanocortin System in IBD: What Evidence?

Studies that have evaluated the melanocortin system in IBD are summarised in Table 2.

The melanocortin system has been extensively studied (although not equally for all its mediators) in major models of experimental colitis, especially murine. The melanocortin system is transparent and strongly imbricated with the hypothalamic–pituitary–adrenal axis [74]. Connections between this axis and IBD have been identified in experimental colitis models [75], so much so that in mice undergoing bilateral adrenalectomy, the course of experimental colitis worsens dramatically [76].

Among the melanocortin members, in the context of IBD, the leading melanocortin, directly and indirectly, is α-MSH. For example, in an experimental murine model of DSS-induced IBD, daily intraperitoneal administration of α-MSH demonstrated, compared to the control, a reduction of blood in the faeces (less than 20% of the control group showed occult blood compared to 100% of the controls at the end of the study). Furthermore, TNF levels were six times lower in patients treated with α-MSH [77]. A further study, like the previous one, also showed the protective effects of α-MSH in models of colic inflammation. In murine nitrobenzene-induced colitis, intraperitoneal administration of α-MSH in a twice-daily regimen for three days showed a reduction in macroscopic lesions (although did not show significance for microscopic lesions), and a protective effect on macroscopic lesions, reversible with selective cyclooxygenase (COX) 1 inhibitor (in the acute colitis group) and selective COX-2 inhibitors (in the chronic colitis group), as well as a reduction in inducible nitric oxide synthase activity [78]. These protective effects have also been shown in preclinical models against endotoxin-induced intestinal damage, ameliorating microscopic and macroscopic lesions [79] and reduced reactive oxygen species produced by rat peritoneal neutrophil granulocytes during inflammation [80].

To search for studies examining melanocortin receptors and primary melanocortin mediators (i.e., α-MSH, β-MSH) in IBD, the following search operators were used:

- MEDLINE: (melanocortin or MC1R or MC2R or MC3R or MC4R or MC5R or α-MSH or β-MSH or alpha MSH or beta MSH or KPV or MSH or melanocyte-stimulating hormone) and (Crohn’s disease or ulcerative colitis or inflammatory bowel disease or IBD or DSS or DNBS or TNBS);
- EMBASE: (“Melanocortin” or “MC1R” or “MC2R” or “MC3R” or “MC4R” or “MC5R” or “α-MSH” or “β-MSH” or “Alpha MSH” or “Beta MSH” or “KPV” or “MSH” or “melanocyte-stimulating hormone”) and (“Inflammatory bowel disease” or “Crohn” or “Ulcerative colitis” or “IBD” or “DSS” or “TNBS” or “DNBS”);
- Web of Science: (all = (melanocortin) or all = (MC1R) or all = (MC2R) or all = (MC3R) or all = (MC4R) or all = (MC5R) or all = (α-MSH) and all = (β-MSH) or all = (alpha MSH) or all = (beta MSH) or all = (KPV) or all = (MSH) or all = (melanocyte-stimulating hormone)) and (all = (inflammatory bowel disease) or all = (Crohn’s disease) or all = (ulcerative colitis) or all = (IBD) or all = (DSS) or all = (TNBS) or all = (DNBS)).

### 4.1. MC1R Mediates and Improves Intestinal Inflammation in Major Models of Experimental Colitis, and Some of Its Agonists Are Being Carefully Studied as Potential Therapeutic Agents in IBD

MC1R, and the pathways it activates, has been mainly studied in experimental colitis models showing how MC1R signalling pathways impact parameters related to colitis disease activity. The gene for MC1R is located on chromosome 16, the same chromosome where one of the genetic susceptibilities to CD has been identified (i.e., that nucleotide-binding oligomerisation domain containing 2/caspase recruitment domain-containing protein 15, also known as NOD2/CARD15) [81,82].

Among the first studies, Maaser et al. [64], in 2006, induced dextran sodium sulphate (DSS)-induced experimental colitis in a group of C57BL/6 mice with a frameshift mutation in the coding sequence for MC1R (MC1Re/e). The authors demonstrated how MC1Re/e and, thus, an impairment in MC1R-mediated signalling resulted in a worse course of DSS-induced experimental colitis in mutated mice than in wild-type (WT) mice. In detail, histologically, after DSS treatment, MC1Re/e mice showed more severe epithelial damage (with more ulcerations and a more pronounced inflammatory cell infiltrate) than MC1R-WT mice. Furthermore, when this model was extended to models of transmissible infectious colitis (i.e., that induced by *Citrobacter rodentium*), it was observed that five days after infection with *Citrobacter*, the number of colony-forming units (CFU) was higher in MC1Re/e mice than in WT, so that at 18 days, WT mice no longer had signs of infection in their faeces, whereas MC1Re/e mice still had signs of CFU in their faeces. All this meant that in mice with an impairment of MC1Re/e signalling, not only was the colitis more severe, but if an infectious strategy had induced it, the level of antimicrobial defence was worse and, consequently, the duration of the colitis phase was prolonged.

Later, Kannengiesser et al. [66] assayed the anti-inflammatory action of a melanocortin-derived tripeptide, i.e., α-MSH(11-14), also called KPV (i.e., Lys-Pro-Val) or 5-phenyl-2-keto-valeric acid [83], in two models of experimental murine colitis: that induced by DSS and that of CD45RB^hi^ mice transfer. This KPV peptide finds its anti-inflammatory rationale in that α-MSH exerts anti-inflammatory actions via the three amino acids 11–14 KPV located at the 3-terminal end [84]. The authors also examined the therapeutic potential of KPV in a third model of DSS-induced colitis in MC1Re/e mice. KPV improved DSS colitis and CD45RB^hi^ mice transfer with an early weight regain compared to control animals and histological findings (e.g., leucitic infiltrate, submucosal oedema, crypt hyperplasia). Finally, an interesting finding was a very different behaviour of KPV in MC1Re/e mice. In detail, KPV could not impact therapeutically as markedly in these classes of mice (i.e., there were no significant weight and histological changes between KPV-treated MC1Re/e mice and controls). However, despite this, the authors recorded a slightly decreased lethality in MC1Re/e mice treated with KPV. This highlights that there are probably MC1R-dependent and -independent mechanisms by which the KPV peptide may act therapeutically in experimental colitis.

The therapeutic potential of KPV in murine DSS-induced experimental colitis was also confirmed in another study in which KPV was administered in nanoparticles [70,85].

It has, finally, been hypothesised that KPV is internalised within intestinal cells via a transporter peptide (i.e., PepT1) [86]. PepT1 is an H^+^-coupled di/tripeptide transporter localised at the apical membrane of intestinal epithelial cells [87]. It can transport H^+^ ions as well as peptides. Its expression is mainly localised at the small intestine level (as well as in the kidney and bile ducts) [87,88,89]. PepT1 does not have constitutively high levels of expression at the colonic level but may increase if inflammatory stimuli are chronically present at the colonic level [90].

Subsequent studies focused on two other MC1R receptor agonists (i.e., PL-8177 and PL-8331). In detail, Spana et al. [71] evaluated the in vitro anti-inflammatory action of PL-8177 and PL-8331 by assessing a reduction in TNF production (by setting ACTH and α-MSH as positive controls) in whole human blood previously stimulated with the bacterial lipopolysaccharide similarly to positive controls. In addition, PL-8177 was administered in mice with experimental colitis induced by dinitrobenzene sulphonic acid (DNBS) administered rectally in Wistar mice. PL-8177 showed comparable activity to the positive control (sulfasalazine) regarding changes in normalised colon weight and the per cent difference in a previously determined histological inflammatory score [91].

More recently, the anti-inflammatory action of PL-8177 was also confirmed in another model of DNBS- and DSS-induced colitis in mice [73]. Specifically, however, the authors also evaluated the pharmacokinetics of PL-8177 after a single oral administration of 70 µg by marking it with ^14^C in dogs, showing a higher concentration in the colic than in the upper gastrointestinal tract. Furthermore, administration in healthy human volunteers eliminated the labelled metabolite mainly via the faecal route (not detected in faeces and urine) without observing any severe adverse reactions.

MC1R was also studied concerning a liposomal nanoparticle system for use in instrumental examinations for IBD. Peñate-Medina et al. [92] evaluated a nano liposomal formulation of α-MSH capable of targeting inflamed intestinal regions by exploiting the abundance of MC1R expression at that level.

Ultimately, MC1R certainly has a role in the pathogenesis of intestinal inflammation in IBD, although the precise mechanism has not yet been fully identified and elucidated.

### 4.2. MC2R Is Involved in the Interaction between UVA and UVB and Murine DSS-Induced Colitis

Hiramoto et al. [69] studied the effect of UVA and UVB on mouse DSS-induced colitis concomitantly (i.e., the mouse was treated daily for five days with both DSS and UVA or UVB). The authors examined how UVA resulted in improvement of the typical symptoms while UVB, on the contrary, significantly worsened the course of DSS-induced colitis. However, the authors also evaluated the expression of MC2R in the colon, which was increased in UVB-treated and not UVA-treated mice. UVB treatment was associated with increased blood levels of ACTH, corticotropin-releasing hormone, urocortin-2, IL-6, IL-18 and histamine while, in contrast, β-endorphin levels were lower in UVA-treated mice. In UVA mice, blood levels of TNF and histamine were also reduced. In addition, the authors observed an improvement in UVB-induced DSS-induced colitis by administering an MC2R inhibitor (which appears to have receptor affinity for ACTH).

### 4.3. Colic Expression of MC3R and MC4R Appears to Differ According to Disease Activity in IBD: Initial Experience and Scarce Evidence

As previously mentioned, MC3R and MC4R are particularly abundant in the central nervous system, which is involved in various inflammatory pathways. However, our research group observed in initial immunohistochemical evidence that MC3R expression (with that of MC4R) was markedly higher in disease-affected CD/UC tracts than in healthy mucosa. The study thus highlighted both the expression of these receptors at the colonic level in patients with IBD and a differential expression proportional to disease activity [72]. 

Indeed, despite the more central localisation of MC4R, its role and peripheral localisations are increasingly emerging. Panaro et al. [93] showed how MC4R is expressed in different portions of the gastrointestinal tract, such as the stomach, small intestine and descending colon. Several cytotypes, such as small intestine cells positive for cholecystokinin, gastric inhibitory peptide, and glucagon-like peptide 1 of mice, showed high expression of the mRNA coding for this receptor. By stimulating it with α-MSH administration, its functions seem related to a paracrine inhibition of electrolyte secretion.

However, the role of MC3R and MC4R in IBD is still mainly unexplored, with markedly scarce evidence. Complex preclinical studies evaluating their expression profiles and mechanistic aspects are still awaited.

### 4.4. Combining Recombinant Bacteria and α-MSH as a Strategy in Experimental Colitis

**Figure 1:**
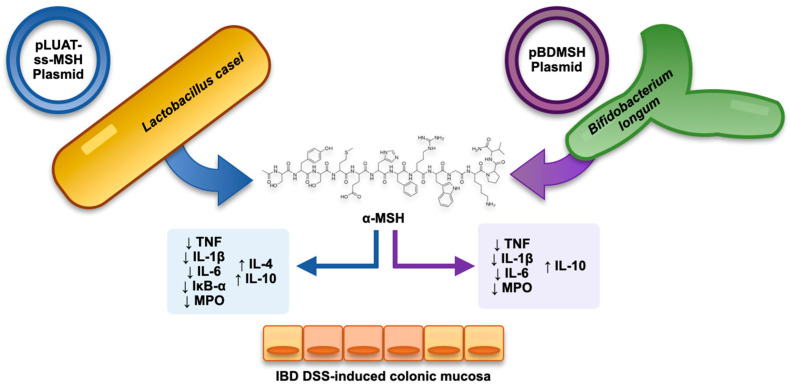
The strategy of α-melanocyte-stimulating hormone (α-MSH) release at the intestinal level in experimental colitis by recombinant bacteria. Two main bacterial strains (i.e., Lactobacillus casei and Bifidobacterium longum) were recombined using two different plasmids (i.e., pLUAT-ss-MSH or pBDMSH) and made α-MSH producers. This strategy was effective in models of dextran sulphate sodium-induced experimental colitis in mice. The anti-inflammatory efficacy was mainly weighed against a reduction in myeloperoxidase (MPO) activity (a harbinger of neutrophilic tissue infiltration and thus reflective of intratissue inflammatory activity) and a modulation of the imbalance between pro- and anti-inflammatory cytokines, as tumour necrosis factor (TNF), interleukins (IL) or nuclear factor of kappa light polypeptide gene enhancer in B-cells inhibitor alpha (IκB-α).

α-MSH has also been studied as a therapeutic agent for experimental colitis by using an intestinal bacterium as the vehicle that delivered it to the colon (Figure 1). Specifically, Wei et al. [94] transformed *Bifidobacterium longum* strains with a specific plasmid (i.e., pBDMSH) to be α-MSH-expressing [68]. The authors then evaluated this recombinant bacterium in DSS-induced murine colitis (after testing the bioactivity of the modified bacterial strain in HT-29 cell models subjected to the inflammatory action of the bacterial lipopolysaccharide). This delivery system of α-MSH (administered orally to mice) was able to improve DSS-induced colitis by regulating the neutrophil infiltrate (as evidenced by modulation of myeloperoxidase) as well as by intervening in the cytokine imbalance by reducing the levels of TNF and IL-1β and IL-6 and increasing the levels of the anti-inflammatory cytokine IL-10. Consequently, the histological activity was also improved under the effect of the recombinant strains. In another study, also conducted in murine DSS-induced colitis, a recombinant strain of *Lactobacillus casei* using the pLUAT-ss plasmid was used to make it α-MSH-producing, with positive results on colitis control (in terms of correcting weight loss, myeloperoxidase and histological activity as well as the survival rate of mice) [65].

### 4.5. Exploiting the Similarity with KPV Tripeptide of the C-Terminal End of α-MSH: The Anti-Inflammatory Role of KPV-like Tripeptide KdPT

**Figure 2:**
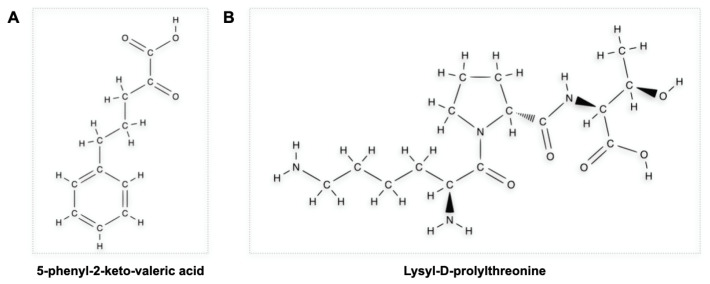
Structure of the tripeptides KPV (**A**), Lys-Pro-Val, and KdPT (**B**), Lys-D-Pro-Thr. The two peptides have been defined several times in the literature as structurally similar. KdPT is homologous to the 193–195 amino acids of interleukin 1β.

The KPV peptide, as already noted, has been the object of numerous studies conducted in the context of experimental colitis. Bettenworth et al. [95] studied a tripeptide structurally similar to KPV (i.e., KdPT, Figure 2) in the context of DSS-induced and IL-10-disrupted mice treated with piroxicam experimental colitis, showing promising potential in attenuating them. In detail, they showed a mechanism by which KdPT reinforced the tight junction between colonic cells. In addition, this peptide did not influence melanogenesis in vitro despite its molecular similarity with KPV.

KdPT is not, de iure, a member of the melanocortin family because its peptide sequence (Lys-D-Pro-Thr) is not found in any member of the melanocortin family, despite being structurally similar to KPV [96]. 

KdPT was previously shown in mice to suppress joint pain by inhibiting IL-1β [97]. This action is hypothesised to be the product of an antagonistic KdPT-IL-1β binding because the L-enantiomer of KdPT is homologous to the amino acid structure of amino acids 193–195 of IL-1β [98,99].

### 4.6. Not Only α-MSH: What Potential of β-MSH?

β-MSH is one of the cleavage products of POMC, consisting of 22 amino acid residues, and is a potent agonist for MC1R, MC3R and MC4R [100,101]. Bradamante et al. [67] tested the efficacy of β-MSH in murine colitis induced by 2,4,6-trinitrobenzene sulphonic acid (TNBS) rectally in Wistar mice. However, the authors also showed substantial variations in efficacy depending on the dosage of β-MSH used, as small or large dosages (i.e., 0.125 and 0.500 mg/kg) did not reduce necrosis of colonic cells. In contrast, an intermediate dosage between the above (i.e., 0.250 mg/kg) dramatically reduced necrosis and improved the macroscopic appearance of the colon. 

### 4.7. Prospects

The use of melanocortin pathways in drug development for IBD is particularly intriguing and exciting, but human experiences are primarily anecdotal. Much of the available evidence is, in fact, preclinical. Despite this, new trials (e.g., NCT05466890) evaluating the safety and efficacy of PL-8177 in active UC are emerging.

Much of the trial evidence relates to other disorders, such as obesity. However, in developing new trials, efficacy profiles must be carefully examined against already available data on safety profiles. Obesity trials conducted with different MC4R agonists have, for example, reported cardiovascular disorders, gastrointestinal disorders (i.e., nausea and vomiting), erectile function disorders (penile erection) or skin hyperpigmentation with different administrations (i.e., oral and subcutaneous) [58].

However, more clinical evidence is available on role of melanocortin receptor modulation as a therapeutic target in immune-mediated inflammatory diseases (IMIDs) other than IBD.

Acthar^®^ Gel (repository corticotropin injection) is a prime example of how the application of a highly purified fraction of ACTH(1-39) [102] has shown significant promise in IMID treatment, showing itself as an adjunctive therapy in rheumatoid arthritis (in patients with inadequate response to glucocorticoids or disease-modifying antirheumatic drugs), in the treatment of patients with persistently active systemic lupus erythematosus as well as in patients with dermatomyositis and polymyositis [103]. This gel, in addition to having a steroidogenic effect (i.e., able to act by promoting steroidogenesis through the MC2R receptor), is also able to exert immunomodulatory effects independently of this pathway (at the level of macrophages and B, T lymphocytes) [104]. In the case of this gel, the safety profile should also be weighed when implementing future IBD studies. Severe adverse events, including infection (i.e., herpes zoster infection even disseminated, chest pain, heart block and avascular necrosis), have also been reported, for example, by one trial investigating this gel in dermatomyositis and polymyositis [105].

It is difficult to track which melanocortin receptor members should be most exploited for new trials in IBD. Nevertheless, it appears that further trials are moving in the direction of MC1R also because of the greater preclinical evidence available. Finally, α-MSH can also represent another potential candidate. In addition, it has already been stressed that α-MSH might represent an excellent example of ‘endogenous-based pro-resolving therapy’ as this molecule, unlike biological drugs directed against a single target, can simultaneously act in the modulation of IL-1β, prostaglandins, TNF, cell adhesion molecules and inflammatory cytotypes such as monocytes, macrophages and neutrophils, as was previously stated [106].

## 5. Conclusions

In conclusion, several melanocortin family members seem interesting and promising when applied to the IBD field. It has been observed that melanocortins certainly have a role in modulating inflammatory processes, and their means of impacting the activity and pathogenesis of experimental IBD have also been shown. There is a clear need for studies evaluating the efficacy and safety of the use of melanocortin metabolites in the therapeutic management of IBD in the clinical setting. 

## Figures and Tables

**Table 1:** Main human melanocortin receptors, ligands, locations and functions.

Receptor	Ligands	Locations	Main Functions
MC1R	Nle-D-Phe47-α-MSH, α-MSH, β-MSH, γ-MSH, ACTH, agouti protein, PL-8177, PL-8331, ACTH(1-39)	Melanocytes, fibroblasts, immune system cells, glial cells, vascular endothelium, melanoma cells	Melanogenesis, inflammation regulation, vascular endothelial regulation, neuroprotection
MC2R	ACTH(1-24), D-Phe47-ACTH(1-24)	Adrenal cortex, adipocytes	Steroidogenesis
MC3R	Nle-D-Phe47-α-MSH, α-MSH, β-MSH, γ-MSH, ACTH, AGRP, SHU9119, ACTH(1-39)	CNS, B lymphocytes, macrophages, placenta, heart, intestine, colon	Energy homeostasis, inflammation regulation
MC4R	Nle-D-Phe47-α-MSH, α-MSH, β-MSH, γ-MSH, ACTH, agouti protein, AGRP, SHU9119, HS014, HS024, setmelanotide, ACTH(1-39)	CNS, colonic mucosa	Energy homeostasis, food behaviour, neuroprotection
MC5R	Nle-D-Phe47-α-MSH, α-MSH, β-MSH, γ-MSH, ACTH, JNJ-10229570, ACTH(1-39)	Ubiquitous	Inflammation regulation, glands secretion

Note: MCR: melanocortin receptor; MSH: melanocyte-stimulating hormone; ACTH: adrenocorticotropic hormone; AGRP: agouti-related protein; CNS: central nervous system.

**Table 2:** Major studies that examined the melanocortin system regarding inflammatory bowel diseases.

First Author, Reference	Year	Main Melanocortin Studied	Inflammatory Bowel Disease Model	Main Results
Maaser et al. [64]	2006	MC1R mutant mice	DSS-induced colitis in C57BL/6 mice and bone marrow chimaeras, *Citrobacter rodentium* murine colitis.	Worse colitis parameters (more severe histological changes and worse weight loss) in MC1R mutant mice. Longer duration of *Citrobacter rodentium* colitis in MC1R mutant mice.
Yoon et al. [65]	2008	α-MSH-expressing recombinant *Lactobacillus casei* strains	DSS-induced colitis	Reduced myeloperoxidase activity, improved histological activity and weight loss.
Kannengiesser et al. [66]	2008	Melanocortin-derived tripeptide α-MSH(11-13) (KPV), MC1R mutant mice	DSS-induced colitis, CD45RB^hi^ transfer colitis	Improvement with KPV treatment of DSS and CD45RB^hi^ colitis regarding weight and histology. Less pronounced improvement in mice with DSS colitis and MC1R-mutated except for lower lethality.
Bradamante et al. [67]	2012	β-MSH	TNBS-induced colitis	Improved macroscopic picture and colonic necrosis of TNBS-treated mice.
Wei et al. [68]	2016	α-MSH-expressing recombinant *Bifidobacterium longum* strains	DSS-induced colitis	Reduced myeloperoxidase activity, TNF, IL-1β, IL-6 downregulation, and IL-10 upregulation. Improved histological activity.
Hiramoto et al. [69]	2016	MC2R	DSS-induced colitis in combination with ultraviolet A or B irradiation	Better outcomes in DSS colitis combined with ultraviolet A irradiation than B irradiation. Increased MC2R expression in DSS-induced colitis plus ultraviolet B.
Xiao et al. [70]	2017	Tripeptide KPV in hyaluronic acid-functionalised nanoparticles	DSS-induced murine colitis	Colonic release of KPV and internalisation in colonic cells with TNF downregulation.
Spana et al. [71]	2019	MC1R agonists (PL-8177, PL-8331)	Human whole blood stimulated by LPS, DNBS colitis Wistar rats	PL-8177 and PL-8331 inhibited TNF levels in whole human blood stimulated by LPS as positive controls (ACTH, α-MSH). PL-8177 improved DNBS colitis as the positive control (sulfasalazine).
Gravina et al. [72]	2022	MC3R, MC4R	Crohn’s disease and ulcerative colitis human specimen	MC3R and MC4R immunohistochemical expression in IBD colonic mucosa (higher in inflamed tracts versus healthy mucosa).
Dodd et al. [73]	2023	MC1R agonist PL-8177	Murine DSS, DNBS colitis, oral PL-8177 pharmacokinetics in rat/dogs, oral PL-8177 in healthy male volunteers (phase 0 study)	DSS and DNBS colitis improvement, colonic bioavailability of PL-8177 in rats/dogs. No systemic circulation of PL-8177 in humans and faecal elimination. No severe adverse events.

Note: MCR: melanocortin receptor; MSH: melanocyte-stimulating hormone; DSS: dextran sulphate sodium; TNBS: 2,4,6-trinitrobenzene sulphonic acid; TNF: tumour necrosis factor; IL: interleukin; DNBS: dinitrobenzene sulfonic acid.
